# Urban heat above and below ground: towards improved understanding, modelling, mitigation and adaptation

**DOI:** 10.1098/rsta.2024.0579

**Published:** 2025-11-06

**Authors:** Alessandro F. Rotta Loria, Jan Carmeliet, Yongling Zhao

**Affiliations:** ^1^ Northwestern University, Evanston, IL, USA; ^2^ ETH Zurich, Zürich, Switzerland

**Keywords:** climate change, cities, urban heat island, subsurface urban heat island, heatwaves, mitigation, adaptation

## Abstract

Heat islands have been recognied at the surface of urban areas since the nineteenth century, while their subsurface counterparts were only identified in the late twentieth century. Since then, surface and subsurface urban heat islands (UHIs and SUHIs), respectively have drawn increasing scientific attention, along with technologies and policies designed to limit their impacts on public comfort and health, infrastructure resilience, the environment and energy efficiency. Yet they have been traditionally studied in isolation. This Theme Issue seeks to bridge that gap by presenting recent advances on *Urban Heat Above and Below Ground*. As the introductory piece of this compilation of works, this article provides an overview of the drivers and impacts of UHIs and SUHIs and offers a perspective on the need to transition towards integrated studies that explicitly account for the thermal interactions between the urban surface and subsurface. Three priority research directions are outlined to address overlooked aspects of urban heat propagation and improve the fidelity of analyses, with a focus on computational simulations. The article concludes by summarizing the contributions in this Theme Issue, which expand knowledge of urban heat dynamics and lay the foundation for capturing the full three-dimensional thermal complexity of cities—above and below ground.

This article is part of the theme issue ‘Urban heat spreading above and below ground’.

## 1. Introduction

The rise of heat islands above and below ground across cities in nearly all climatic zones worldwide [1,2], often referred to as urban heat islands (UHIs) and subsurface urban heat islands (SUHIs), respectively, have emerged as one of the most pressing challenges of our time. Historically, UHIs were first documented in the early nineteenth century in London [3], whereas systematic evidence of SUHIs only began to emerge in the late twentieth century in Tokyo [4]. Since then, both UHIs and SUHIs have been recognied as endemic features of the built environment: as soon as cities are constructed and operated, heat is inevitably released above and below ground, regardless of the materials or technologies employed. However, UHIs and SUHIs have been customarily analysed in silos by either focusing on the physical processes that drive these phenomena above or below the ground.

Traditionally, research on UHIs has been undertaken by urban climatologists, building physicists, energy engineers, fluid dynamicists, environmental engineers, transportation engineers and public health scientists, whereas investigations of SUHIs have largely involved geoscientists, civil and environmental engineers, energy engineers and public health scientists. Consequently, studies of these phenomena have been published in disparate journals and debated within distinct scholarly communities, leading to a fragmented state of knowledge.

In reality, UHIs and SUHIs are coupled, and interact with each other through the continuous flow of heat (and mass, like air and water) above and below the ground, depending on the local thermodynamic conditions. These interactions between UHIs and SUHIs take place within a physical region of finite thickness. The little evidence available about the interplay between UHIs and SUHIs has shown that their coupling and interactions are particularly evident up to a height above the ground surface of a few hundred metres [5] and down to a depth of at least 0.5 m [6]. However, knowledge on the properties of this *interaction zone* developing both above and below the ground remains extremely limited. Addressing the multiple challenges of urban heat spreading above and below the ground demands a sophisticated, integrated approach that seamlessly combines reliable and accurate modelling and prediction, data-driven urban planning, green technological innovation and concrete policy making, while prioritiing social equity and economic sustainability [7,8]. Only in this way can we address the multifaceted impacts of UHIs and SUHIs while supporting global urban populations—and particularly vulnerable groups such as low-income communities and older adults, who are often disproportionately affected due to limited access to cooling resources and residence in heat-prone, low-vegetation neighbourhoods [9,10].

This Theme Issue aims to provide a distinct contribution to the state-of-the-art by gathering in a unique forum 16 studies on UHIs and SUHIs, encompassing experimental investigations, computational analyses, an opinion, and the present perspective. As the Introductory Article to this compilation of studies, this perspective work (i) revisits the physical processes and drivers underlying UHIs and SUHIs; (ii) summaries their key impacts; (iii) provides a critical analysis of prevailing investigation approaches, with a focus on computational studies; (iv) formulates distinct yet interconnected research questions to advance the understanding, modelling, mitigation and adaptation of urban heat within the three-dimensional complexity of cities; and (v) offers closing considerations that open this Theme Issue, ultimately serving as a bridge between the current state of knowledge on urban climates above and below ground and the opportunities ahead.

## 2. Physics of surface and subsurface urban heat islands

### (a) The problem

**Figure 1.**
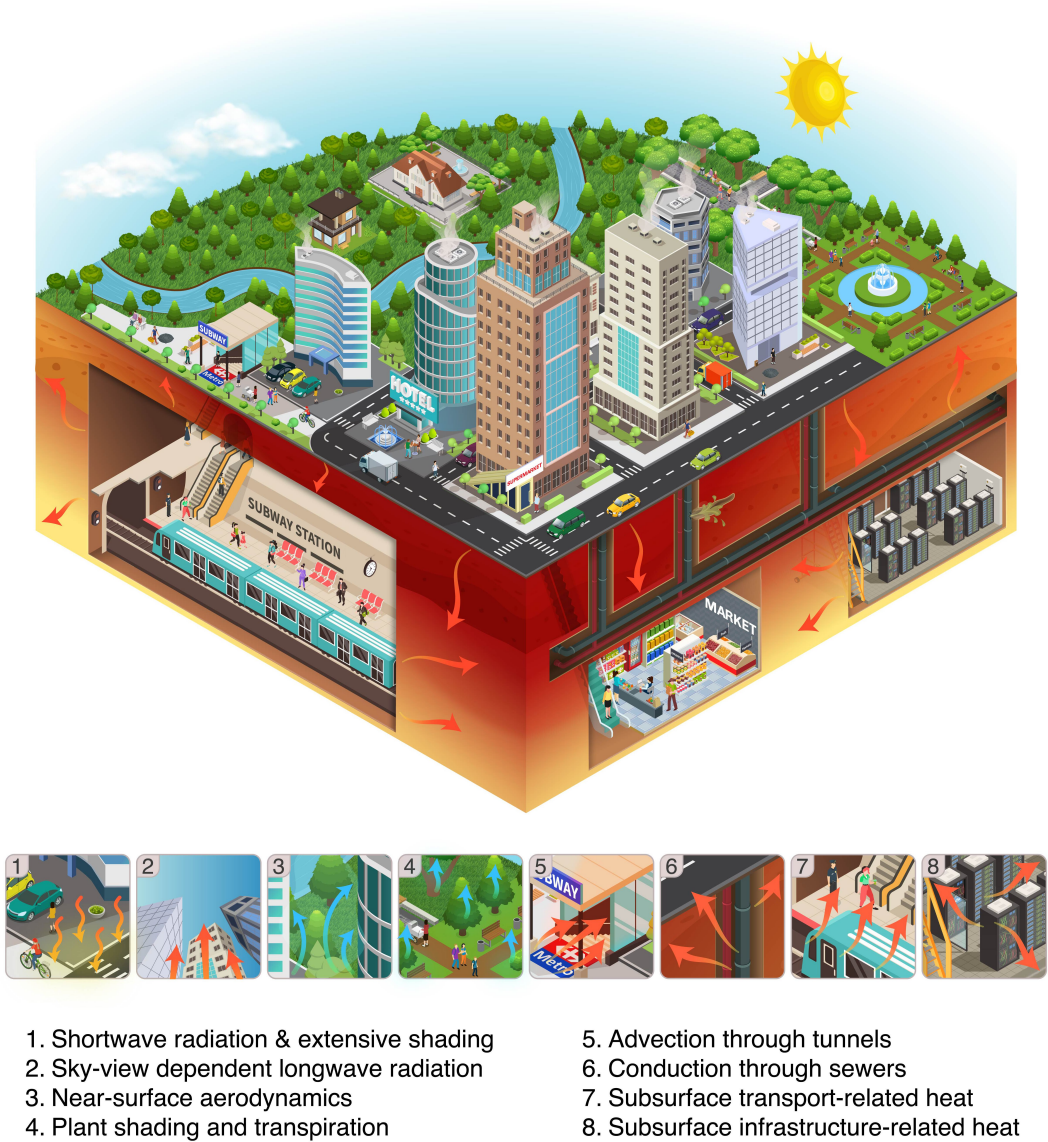
The spread and coupling of urban heat above and below ground. The infographic presents selected heat and mass transfer processes among the myriad ones that drive UHIs and SUHIs.

UHIs and SUHIs are highly complex phenomena, which spread above and below the ground across urban forms through the coupling of multiple physical processes, as illustrated in figure 1. Despite this complexity, UHIs and SUHIs are governed by three basic modes of heat transfer: radiation, conduction and convection. Latent heat transfer, typically intertwined with convection, can also occur through evaporation from ground moisture and water bodies, as well as transpiration from plants, playing a critical role in modulating surface and atmospheric temperatures within urban environments [7,11].

The relative contribution of radiation, conduction and convection to the intensity of UHIs and SUHIs (a distinct metric used to quantify the severity of UHIs and SUHIs through the difference between a relevant temperature in the studied urban area and a reference temperature in a neighbouring rural area) depends on global climatic forces and localied urban dynamics features that develop across highly variable spatial and temporal scales, and are in turn, influenced by the morphology, geometry and site-specific attributes of the built environment under consideration. Similarly, the coupling and interactions between UHIs and SUHIs are set to vary on a case-by-case basis, being particularly pronounced within the interaction zone.

### (b) Diffused drivers of UHIs and SUHIs

The principal diffused driver of both UHIs and SUHIs is global warming [12]. Rising atmospheric temperatures, primarily due to increased greenhouse gas concentrations, directly affect the thermal environment at the surface of cities through enhanced longwave radiation from the atmosphere and heat conduction, storage and successive release from the materials that constitute the urban surface. Indirectly, rises in surface air temperature also influence the thermal environment beneath cities, as a fraction of heat is set to diffuse from the surface to the subsurface depending on the local thermal conditions [13], primarily through conduction but potentially also via advection as a result of groundwater flow or airflow within underground built environments.

Another diffused driver of UHI and SUHIs consists of extreme heat waves, which can cause acute and sustained spikes in air and ground surface temperatures [14,15]. In fact, extreme heat can exacerbate the amount of heat accumulating in cities, with dire consequences for the thermal environment characteriing the urban surface and, in turn, the urban subsurface.

Hydrodynamic flow processes serve as additional diffused drivers—or, in some cases, mitigators—of urban heat accumulation. At the surface, airflow patterns driven by wind and local urban geometry modulate the spatial distribution of sensible heat through convection, redistributing warm or cool air masses across different urban zones [16,17]. In areas with poor ventilation, stagnant air masses allow heat to build up, increasing surface and near-surface temperatures. In the subsurface, groundwater flow plays a parallel role through advection, transporting heat away from or towards specific areas [18]. Similarly, sea-level rise can elevate groundwater tables in coastal cities, altering subsurface flow regimes and enhancing the horizontal and vertical redistribution of heat [19].

Importantly, while global warming, heat waves and hydrodynamic flow processes are not exclusive to cities, their interactions with urban geometry and infrastructure lead to uniquely intensified thermal responses in urban environments. Distinct intensification of UHI due to the synoptic forcing has been seen in Sydney, Paris and most Chinese cities [20–22], though the impact tends to be unclear on SUHIs. Their effects are widespread and pervasive, qualifying them as diffused drivers of both UHIs and SUHIs. These processes—mediated by radiation at the surface, convection in the air and water domains and conduction through solid materials—jointly shape the spatiotemporal dynamics of heat accumulation in cities, making them critical components in understanding and mitigating urban thermal risks.

### (c) Localized drivers of UHIs

UHIs are shaped by a complex interplay of physical, structural and anthropogenic drivers that locally unfold across the urban fabric. While their manifestation typically encompasses entire cities, the actual drivers of UHIs operate at very local scales, sometimes varying within a single city block [15,23]. In some cases, localied physical processes associated with specific drivers can even dominate the broader spatial expression of urban warming [7]. Despite the inherent complexity and spatial variability of UHIs, their emergence can be broadly traced to two overarching phenomena: direct heat storage in urban surfaces and heat accumulation in the urban canopy layer.

A fundamental mechanism driving UHIs is the way urban surfaces interact with solar and terrestrial radiation. Materials commonly used in cities—such as asphalt, concrete and dark roofing—typically exhibit low reflectivity and high thermal mass [24]. These properties enable them to absorb large quantities of incoming shortwave solar radiation during the day and re-emit stored energy at night as longwave radiation, thereby sustaining elevated near-surface temperatures long after sunset. The net result is that the heat storage component of the surface energy balance is considerably larger in urban settings than in rural ones. During daytime hours, heat storage in urban environments can account for 17 to 58% of the net all-wave radiation [25], reflecting a significant portion of solar energy retained within built surfaces [15,26]. Reviews of the thermal behaviour of urban pavements, focusing on their resulting cooling abilities, have been reported by Anand *et al*. [27] and Seifeddine *et al*. [28]. Impervious surfaces further disrupt the urban moisture balance. By sealing the ground and preventing water infiltration, they suppress evaporative cooling and limit latent heat fluxes, which would otherwise reduce surface temperature [29–31]. The reduction in latent heat exchange increases the partitioning of available energy into sensible heat, which is then transferred both vertically and horizontally via convective and advective processes in the atmosphere [26,32]. Stormwater systems that swiftly channel rainwater away exacerbate this issue by reducing opportunities for surface wetness and evaporative cooling.

Compounding this is the scarcity of vegetation in many urban areas. Without trees and green spaces—natural agents of cooling through evapotranspiration and radiative shading—urban surfaces retain more heat [33–35]. Vegetative cover also alters the surface energy budget by reflecting solar radiation and reducing net radiative gain. A tree cover of approximately 16% is found to lead to an average 1°C drop in urban surface temperature, and 32% for a 2°C drop, in European urban areas [36]. In 110 cities across 17 climates, the implementation of trees reduces peak monthly temperatures to below 26°C in 83% of the cities [35]. At the local scale, when present, greenery can significantly alter microclimates; for instance, shading from urban trees has been shown to reduce pedestrian-level temperatures by up to 12°C in cases with large radiation blockage and transpiration [35].

Above the surface, within the volume of air bounded by buildings—the urban canopy layer—waste heat accumulates because of both radiative trapping and limited convective ventilation. Key factors include the sky view factor, street orientation, building height and spacing [37,38]. Together, these elements determine how effectively heat is radiated back into the atmosphere and how well air can circulate. Narrow streets flanked by tall buildings reduce sky exposure, limiting longwave radiation loss at night and effectively trapping heat close to the ground. The urban form also affects airflow [39–41]. Dense developments and enclosed street canyons hinder wind movement, reducing the ventilation capacity of the urban canopy [42]. Poor ventilation creates stagnant air masses where heat accumulates, particularly during calm, clear nights [43,44]. Even small increases in building density or height can significantly amplify UHI intensity. For example, a hypothetical 25% increase in building volume in Berlin, achieved by modifying either building height or urban fraction, has been shown in numerical experiments to raise UHI intensity by approximately 0.2°C and 0.4°C, respectively [45]. Therefore, the urban morphology controls both radiative heat retention and convective heat dispersion, modulating the overall thermal performance of the canopy layer. The building frontal area, orientation and spacing all contribute to shaping local microclimates by adjusting the balance between heat absorption, storage and removal [45–47].

Adding to this urban thermal burden is the direct emission of heat from human activities, primarily transferred to the atmosphere via convection. Buildings release waste heat through heating, ventilation and air conditioning systems, vehicles emit heat through combustion and industrial activities act as continuous thermal sources. These inputs elevate air temperature, with effects that vary as a function of the surface characteristic heterogeneity—including variations in albedo and the distribution of pervious versus impervious surfaces, which together define the local energy budget and the capacity of the surface to absorb or reject heat [48].

### (d) Localied drivers of SUHIs

Similar to UHIs, SUHIs are shaped by a complex interplay of physical, structural and anthropogenic drivers that locally unfold across the underground urban fabric. While the phenomenon typically characteries the subsurface of entire cities, its genesis is primarily and inherently local, shaped by spatially and temporally variable interactions between the built environment and the ground [19]. As the subsurface counterpart of UHIs, SUHIs emerge from two overarching phenomena: direct heat storage and waste heat accumulation in the urban subsurface.

The subsurface of cities is increasingly saturated with anthropogenic heat, stemming from human activities that generate and reject thermal energy into the ground [49]. Buildings with conditioned basements, underground parking structures and utility corridors dissipate heat into surrounding ground through their foundations and envelope surfaces [50–52]. The extent of heat transfer is influenced by insulation levels, occupancy patterns and energy usage within the structures. Particularly in older buildings or infrastructure lacking thermal insulation, continuous heating and cooling cycles translate into sustained thermal loading of the ground. In addition to building basements, which often represent the most substantial localied driver of SUHIs [53,54], buried transportation infrastructure greatly contributes to heat losses in the urban underground [55]. Even air-conditioned underground stations and transit lines function as point sources of heat, with energy dissipated via walls, floors and ventilation systems into the surrounding geology [56]. Energy infrastructure, such as district heating pipelines, high-voltage cables and sewage lines, further generates waste heat [57]. For example, uninsulated hot water mains can raise ground temperatures by several degrees in their immediate vicinity, creating thermal plumes that slowly migrate through the subsurface over time, as observed for geothermal systems [58] and underground thermal energy storage systems [59]. Importantly, the cumulative and continuous nature of these heat inputs differentiates SUHIs from their atmospheric counterparts. Once heat enters the ground, the low thermal diffusivity of soil and rock dictates that this energy is retained over long periods and spreads slowly [18]. This allows anthropogenic heat sources to imprint long-lasting thermal anomalies in the subsurface [51], often persisting and aggravating over multiple decades.

Beyond anthropogenic heat sources, the physical structure and composition of the subsurface play a crucial role in shaping how and where heat accumulates underground [60]. The heterogeneous distribution of soil and rock, groundwater presence and velocity, and artificial alterations from excavation and construction activities lead to a spatially uneven underground thermal environment. Urban excavation practices create a patchwork of subsurface voids, backfilled zones and compacted layers, often with thermal properties significantly different from that of the native ground. Fill materials such as rubble, crushed stone or construction waste typically exhibit lower moisture retention and higher thermal conductivity, facilitating faster heat diffusion near the surface but also reduced long-term storage [18]. Conversely, natural soils and saturated sediments can act as thermal capacitors, absorbing and slowly redistributing heat [18]. This interplay determines the vertical and horizontal spread of thermal anomalies beneath cities. The geometry of underground structures—depth, orientation, and continuity—also controls the magnitude and propagation of waste heat. For instance, long underground corridors or foundation slabs can act as horizontal radiators, spreading heat away from point sources. In addition, the presence of groundwater introduces a dynamic component: advection can transport thermal energy over larger distances, creating asymmetric heat plumes that depend on flow direction and velocity. In areas with low permeability or stagnant water tables, however, conductive processes dominate, leading to localied and persistent warming zones. Moreover, the thermal stratification of the urban underground—wherein shallow layers are significantly warmer than deeper strata—can establish gradients that drive slow vertical migration of heat, influencing both the behaviour of soil and rock, and the thermal regime experienced by buried infrastructure [61].

### (e) Couplings and interactions between UHIs and SUHIs

UHIs and SUHIs are thermally interconnected phenomena that emerge from a dynamic interplay of conduction, advection and radiation. These physical processes govern the exchange, redistribution and persistence of heat across the urban surface and its underlying ground, giving rise to complex thermal patterns that evolve over time and space [62].

Conduction is the most direct and widespread heat transfer process linking UHIs and SUHIs. Elevated surface temperatures—driven by solar absorption, impervious materials and anthropogenic heat emissions—create a vertical thermal gradient that propels heat downward into the ground. As this gradient persists, heat slowly diffuses through the upper soil and rock layers, warming the subsurface with depth over time. The rate of conduction is modulated by the thermal conductivity of surface and subsurface materials, which is, in turn, influenced by moisture content and density. Dry, loosely packed soils retard heat flow, while fully or partially saturated soils, often found near water mains or in low-lying zones, conduct heat more effectively and allow it to penetrate deeper into the ground. These variations influence how heat is transferred in the subsurface: daily temperature shifts typically affect the top 5−20 cm of soil [63], while seasonal patterns can extend to depths of 1−3 m [64].

Beyond this vertical pathway, advection enhances the global heat transfer process characteriing cities through the action of a fluid in motion, which may occur above and/or below ground. At the surface, airflow within the urban canopy layer redistributes heat through convection, carrying warm air from heat-intensive zones towards adjacent areas and potentially altering the thermal load on the ground. These air movements are shaped by urban morphology—building heights, street orientations and sky view factors—which either promote or suppress ventilation. In poorly ventilated zones, reduced convective cooling allows surface temperatures to climb, increasing conductive heat input to the subsurface. Below ground, groundwater flow serves as a potent advective force, transporting heat away from its sources through convection. In permeable soils or aquifers, thermal plumes originating from heated basements, buried infrastructure or energy systems can be carried over significant distances by horizontal groundwater movement [50,65]. This process not only extends the spatial reach of SUHIs beyond areas of direct surface influence but also contributes to the development of persistent and mobile subsurface thermal anomalies.

In addition, radiation governs how heat is exchanged between urban surfaces and the atmosphere, influencing both surface and subsurface thermal dynamics. During the day, surfaces absorb shortwave solar radiation, with dark, low-albedo materials converting much of it into stored thermal energy. At night, longwave radiation becomes dominant, as surfaces release heat back into the atmosphere. However, this cooling process is often suppressed in urban environments. Narrow streets and tall buildings reduce the effective sky view, trapping longwave radiation and limiting radiative losses. The result is that heat retention is prolonged at the surface and, by extension, in the subsurface. Moreover, radiative energy not dissipated to the atmosphere can further enhance conductive fluxes into the ground.

The surface and subsurface do not operate as isolated systems, but rather as a vertically integrated thermal continuum, where energy is constantly exchanged, redistributed and sometimes amplified by the spatial configuration and infrastructure of the city itself. Understanding these coupled heat transfer processes is essential for predicting urban thermal behaviour and designing strategies that mitigate the compounding effects of surface and subsurface warming.

These localied drivers for a surface–subsurface integrated volume can be generalized and described below, following a heat and moisture balance perspective:


(2.1) $$R_{n}+A^{z}=H+G+L+F_{ad}^{z},$$

(2.2) $$M_{n}=E^{z}+T+P+M_{an}^{z}.$$

In equation (2.1): $R_{n}$ denotes net all-wavelength reflective heat; $A^{z}$ denotes anthropogenic heat that directly contributes to UHIs or SUHIs (where z refers to UHI or SUHI), originating either within the urban canopy layer or beneath the ground surface; H and L represent ground surface sensible and latent heat, respectively; G denotes heat storage in the surface–subsurface interaction zone; and $F_{ad}^{z}$ denotes advection-associated heat transfer both above and below the ground, such as through metro tunnels. In equation (2.2): $M_{n}$ represents the net moisture; $E^{z}$ denotes evaporation-associated moisture change generated on and below the ground surface; T denotes plant transpiration-associated moisture change; P denotes precipitation-induced moisture variation; and $M_{an}^{z}$ denotes moisture change due to anthropogenic processes occurring on and below the ground surface, such as humification from buildings and underground systems. Note that here $E^{z}$ can be associated with both evaporation from water bodies and routine urban irrigation in the urban canopy layer, as well as the drying processes beneath the ground surface.

## 3. Impacts of surface and subsurface urban heat islands

### (a) General

As a result of their complex driving forces, UHIs and SUHIs create a dual crisis. Above ground, UHIs can dramatically elevate urban temperatures by up to 10°C compared with surrounding rural areas [66]. Below the surface, SUHIs can generate can generate localied hotspots with anomalies exceeding 25°C relative to nearby reference conditions [52]. The impacts of these phenomena intensify during heat waves [67] and extend across public health, infrastructure resilience, environmental sustainability and energy efficiency [11].

### (b) Impacts on public comfort and health

The public comfort and health implications of urban thermal anomalies, encompassing both UHIs and SUHIs, manifest primarily through elevated rates of morbidity and mortality in urban populations [68–70]. Cities in lower latitudes often experience an increase in mortality [71]. Heat exposure, when accounting for population, in only 25 high-risk countries—including India, Bangladesh and Pakistan—contributes to nearly 25% of the global impact. Epidemiological research also reveals a marked disparity between urban and suburban health outcomes, with urban environments demonstrating significantly higher incidence of heat-related health complications [72]. This urban–suburban differential is particularly noteworthy in its magnitude, as quantitative analyses indicate that urban residents face greater heat-related health risks compared with their suburban counterparts. The distribution of these health impacts exhibits considerable spatial variation within urban environments, with socially vulnerable populations—particularly older adults and individuals with underlying medical conditions—experiencing disproportionate effects [73]. This spatial heterogeneity in health outcomes correlates strongly with local thermal patterns, as neighbourhoods experiencing more intense UHI effects typically report higher rates of heat-related illness [72].

The health burden becomes especially pronounced within underground transportation infrastructure, particularly in subway systems, where thermal management presents unique challenges [74]. Legacy transit systems, such as London, New York, Boston and Paris, characteried by ageing infrastructure and limited ventilation capabilities, create environments conducive to severe heat-related medical conditions, including thermal stress, fluid imbalance and associated physiological complications [75]. The thermal dynamics in these underground spaces is further complicated by thermodynamic processes such as buoyancy-driven airflow, where temperature differentials drive vertical air movement through the station infrastructure. This phenomenon intensifies the thermal burden on both infrastructure systems and transit users, creating potential public health concerns that require targeted intervention strategies [75–77].

### (c) Impacts on the environment

The environmental ramifications of UHIs extend across multiple ecological domains, with marked effects on atmospheric chemistry [78], meteorological patterns [79] and ecosystem functioning [80]. The atmospheric environment experiences substantial modification through enhanced photochemical processes, leading to altered pollutant transformation and transport mechanisms. These modifications manifest in the urban boundary layer through disrupted precipitation regimes and perturbed hydrological cycles, primarily attributed to intensified evaporative processes and modified water vapour distribution [81]. The alteration of local wind fields and atmospheric circulation patterns further compounds these effects by modifying urban ventilation characteristics, thereby creating feedback mechanisms that influence local environmental quality. These atmospheric perturbations subsequently influence urban ecological systems, as evidenced by modified phenological patterns and shifts in urban biodiversity composition [82].

The environmental alterations caused by SUHIs, while less readily observable, present equally significant concerns with potentially more extensive long-term implications [83]. These underground impacts encompass modifications to soil ecological processes, including shifts in microbial community dynamics and biogeochemical cycling [84]. The vertical thermal profile of urban soils exhibits persistent modifications, leading to cascading effects on groundwater quality [85]. Although these subsurface thermal anomalies may not demonstrate immediate visible consequences, their cumulative effects on urban ecosystem functioning and environmental sustainability warrant particular attention in urban environmental management strategies [6,86–89]. The interconnected nature of surface and subsurface processes suggests that comprehensive approaches to urban thermal management must consider both domains simultaneously.

### (d) Impacts on infrastructure

The thermal stresses and deformations induced by UHIs pose significant challenges to contemporary urban infrastructure, particularly because existing systems were largely designed without consideration for intensified urban thermal loading [90]. This challenge is most evident in renewable energy systems, where performance is inherently temperature-dependent. For instance, the efficiency of photovoltaic installations decreases markedly under elevated operating temperatures [91]. Surface transportation infrastructure also demonstrates notable vulnerability, as thermal expansion and contraction cycles accelerate the deterioration of pavement materials, necessitating more frequent maintenance interventions [92]. These thermal impacts manifest differently across various infrastructure types but consistently result in accelerated material degradation and increased operational challenges.

The implications become particularly acute when considering the effects of SUHIs on underground infrastructure, including building foundations, tunnels, underground parking garages and subterranean utilities [19]. Subway systems frequently experience extreme platform temperatures due to limited ventilation, which not only affect passenger comfort but also strain mechanical and electrical systems [74]. These combined effects lead to several concerning outcomes: accelerated deterioration of construction materials, increased frequency and costs of maintenance operations, and shortened infrastructure lifespans. As climate change continues to exacerbate urban warming, the adoption of thermally adaptive design principles becomes crucial for infrastructure resilience [93]. This necessitates a fundamental shift in infrastructure design philosophy, incorporating both immediate heat mitigation strategies and long-term climate adaptation measures in urban infrastructure planning and management. The foundations that support our cities were also not designed with specific consideration of temperature anomalies associated with SUHIs, which are causing the ground to deform [12]. These ground deformations can be problematic for the durability of foundation systems and other underground constructions, depending on the technological features of the earth-contact structure under consideration and its overlying structure(s) [19,94]. Site conditions also play a major role in determining the effects of SUHIs on underground constructions. Evidence indicates the heat accumulated underground by SUHIs has altered ground stiffness, extending progressively deeper into the subsurface and resulting in reduced permeability and variability in other key engineering properties, which can involve unwanted impacts on infrastructure integrity [95].

### (e) Impacts on energy systems

The thermal anomalies associated with UHIs manifest complex implications for urban energy systems, primarily through modifications to consumption patterns and infrastructure performance characteristics [96,97]. Direct energy consumption exhibits significant perturbation, with empirical evidence indicating that cooling demand in urban cores can exceed suburban requirements by up to several times, such as in urban and rural London [96]. This amplified energy demand creates cascading effects on infrastructure systems, where power generation and distribution networks experience compound challenges: thermal power plants demonstrate reduced efficiency under elevated ambient temperatures, while transmission infrastructure faces increased thermal loading. The combination of heightened demand and compromised system efficiency creates particular vulnerability during peak demand periods, potentially leading to critical infrastructure stress points in urban energy systems [98]. These primary effects are further complicated by feedback mechanisms that create self-reinforcing cycles of urban warming and energy consumption [99]. The anthropogenic heat released through increased cooling system operation contributes additional thermal loading to the urban environment, potentially increasing local temperatures by more than 1°C during peak cooling periods [100]. This creates a complex coupling between urban climate and energy systems, where increased cooling demand generates additional waste heat, further exacerbating the UHI intensity and creating subsequent increases in cooling requirements. This feedback loop presents challenges for urban energy planning, as traditional linear approaches to infrastructure capacity planning may inadequately address these compound effects. The interaction between surface and subsurface thermal regimes further complicates these dynamics, necessitating revised approaches to urban energy system design and management.

Fortunately, SUHIs also present a powerful opportunity for urban energy systems. On the one hand, SUHIs encourage the renewal of existing building stock and the construction of new buildings and infrastructures by incorporating architectural and engineering solutions to create more efficient and sustainable spaces with adequate thermal insulation [12]. Clearly, neither the retrofit of existing buildings nor the construction of new buildings would allow to zero the diffusion of heat from constructions to the underground, especially the heat deriving from anthropogenically induced exothermal processes (e.g. cement hydration reactions, heat losses from machinery or other utilities and facilities) or sealed urban surfaces (e.g. asphalt and concrete), which are recognied to also contribute to SUHIs [56,57,88]. However, these practices could mitigate the current intensity of SUHIs. On the other hand, SUHIs strengthen the rationale for deploying shallow geothermal technologies, which can transfer the heat driving underground climate change from the ground to buildings and infrastructures, which are massive thermal energy consumers [65,101–103]. Developing geothermal technologies should be considered a secondary measure in any strategy aimed at mitigating SUHIs, as the most effective approach involves addressing the issue at its source through thermal insulation of underground building enclosures. However, in cases where retrofitting is impractical, impossible or uneconomical, the deployment of geothermal technologies across urban areas presents a compelling and economically viable alternative, with the additional advantage that they exhibit an enhanced heat extraction capacity due to the presence of waste heat [104]. In fact, findings suggest that for every degree of ground temperature anomaly, the size of geothermal technologies such as boreholes can be reduced by approximately 4 m while maintaining the same power output [105]. Notably, traditional shallow geothermal technologies have customarily required boring of the ground to access geothermal heat, but recent innovations have overcome this challenge [18], addressing a key barrier to the widespread deployment of geothermal systems in urban environments.

## 4. The role of surface–subsurface couplings in future urban climate studies

### (a) A critical analysis of current modelling approaches

Despite significant progress in the analysis of UHIs and SUHIs, and their impacts, contemporary investigation approaches remain largely segmented, with most efforts focusing independently on either surface or subsurface domains and related processes. This compartmentaliation limits our capacity to fully understand the complex thermal interactions that define the spread of heat across urban environments above and below the ground, and above all, their intricate impacts. Although experimental studies can be designed to quantitatively assess the interactions between UHIs and SUHIs, computational approaches probably represent a lower-hanging fruit for addressing this challenge, with direct implications for improving understanding, mitigation and adaptation. Based on these considerations, an analysis of challenges and opportunities arising in this scope is presented hereafter with reference to table 1.

**Table 1.** Key physical processes, models and uncertainties for urban microclimate modelling.

	physical processes	modelling	uncertainty
UHI	solar shortwave radiation	position of the sun	impact of cloud
	radiation absorption, heat dissipation and storage on building surfaces	building surface heat flux budget	heterogeneity of building materials and thermal properties
	moisture transport	wind-driven advection and turbulent diffusion	quantification of plant transpiration and large water body-related moisture source
	urban aerodynamics	Reynolds-averaged Navier–Stokes and large-eddy simulation models	geometrical representation of aerodynamics-relevant urban forms
	sensible and latent heat fluxes transport in the air domain	advection–diffusion	representation of the stability of urban boundary-layer flow
	radiation-blocking induced shading	sun path determined direct and diffuse radiation	resolution of sky view factors
	vegetation transpiration	Penman–Monteith transpiration equation	stomatal conductance models and characterization of vegetation types and growth
	water evaporation	Penman equation	characterization of urban major water bodies
	anthropogenic heat	localized heat source	spatial and intensity heterogeneity of anthropogenic heat
surface-subsurface interface | interaction zone	heat flux budget at the interface	surface energy budget	temporal and spatial resolution of heat storage
	moisture budget at the interface	Richards' equation or empirical infiltration models	characterization of soil types and conditions in cities
SUHI	heat transfer in ground	heat conduction (and advection in the presence of groundwater flow)	soil and rock type-specific, moisture content-dependent thermal properties
	moisture transport in ground	seepage flow in porous media	water retention and hydraulic conductivity
	anthropogenic heat	source term in energy conservation equation, or direct resolution of global problem	representation of heat emissions from underground infrastructure
	plant root water uptake	sink term in Richards' equation	site-specific water retention and hydraulic conductivity data

One of the most prominent limitations of the state-of-the-art lies in the representation of the coupling between the heat and mass transfer processes occurring across the above- and below-ground domains of cities—what may be termed surface–subsurface coupling within the interaction zone. Nominally delimited by the physical interface of the ground surface, these processes depend on the properties and conditions of a broader zone extending both above and below this interface, rather than being confined to a simple one-dimensional layer. Established modelling practices typically impose oversimplified boundary conditions at the ground–atmosphere interface, assuming spatially uniform surface properties such as albedo, thermal conductivity, specific heat and density. However, real urban surfaces are highly heterogeneous, composed of diverse materials, including asphalt, concrete, soils and vegetation, each exhibiting unique and non-uniform thermo-physical properties. Furthermore, the spatial complexity introduced by urban geometry—building shadows, varying surface roughness and transient solar exposure—generates non-uniform radiative forcing patterns that are rarely resolved at appropriate scales. As a result, the heat budget of the inhomogeneous region close to the ground surface, which governs the heat transfer between the surface and subsurface, is often mischaracterized. The inability of current models to capture daily and seasonal hysteresis effects, dynamic heat storage and the role of shallow geothermal gradients further exacerbates these limitations. In alignment with these considerations, evidence shows that boundary conditions can largely affect modelling results [106–111], yielding unwanted inaccuracies.

Another critical shortcoming lies in the treatment of rainfall, groundwater content and their dynamics in analyses of UHIs and SUHIs. The thermal properties of soil and rock—particularly thermal conductivity and volumetric heat capacity—are highly sensitive to water content. Nevertheless, many models either neglect moisture variability or apply static parameterizations that fail to capture real-time hydrological fluctuations. This oversimplification can introduce substantial errors, particularly in humid or semi-arid environments where ground moisture exhibits strong temporal and spatial variability. Current urban climate vegetation models also often fail to adequately account for the effects of ground moisture on root systems and accurate transpiration. Furthermore, the interaction between moisture and temperature fields is inherently nonlinear, influencing processes such as latent heat flux, evaporation and thermal buffering—mechanisms essential for accurately simulating UHI and SUHI intensity and persistence.

An additional limitation of current modelling approaches consists of the widespread employment of unrealistic boundary conditions to represent urban wind dynamics. Studies in the field have to rely on wind data from meteorological stations. However, these stations are typically located outside urban areas or in parks, thereby providing only partially representative information of the conditions that truly characterie the modelled urban areas. Some simulation tools, such as ENVI-MET, provide different forcing models to address this issue to some extent. A reliable approach may be relying on downscaling using nested models to address the issue [112,113].

Equally approximate is often the simulation of surface and subsurface sources of anthropogenic heat. These heat sources exhibit complex spatial and temporal patterns, varying by the specific technological type and features of the heat source, time of day and seasonal factors. However, comprehensive data characteriing these features remains largely unavailable for modelling purposes, highlighting the need for systematic data collection and accessible online databases. As a result, not only are anthropogenic heat sources often modelled in an approximate manner based on limited data, but they are frequently omitted altogether—particularly surface anthropogenic heat sources in models addressing SUHIs, and subsurface anthropogenic heat sources in simulations focused on UHIs.

The parameterization of material properties in both surface and subsurface layers also require considerable refinement. While three-dimensional representations of urban morphology have become increasingly accessible, models continue to rely on simplified or idealized thermal properties for key materials. For example, pavement and building materials are typically characteried using laboratory-derived values that do not account for real-world ageing, surface degradation or material mixing. Similarly, the thermal behavior of urban vegetation—shaped by canopy geometry, species-specific transpiration rates, irrigation regimes and phenology—is rarely represented with sufficient detail, despite its critical influence on shading, cooling and overall energy balance.

### (b) The pathways forward

**Figure 2.**
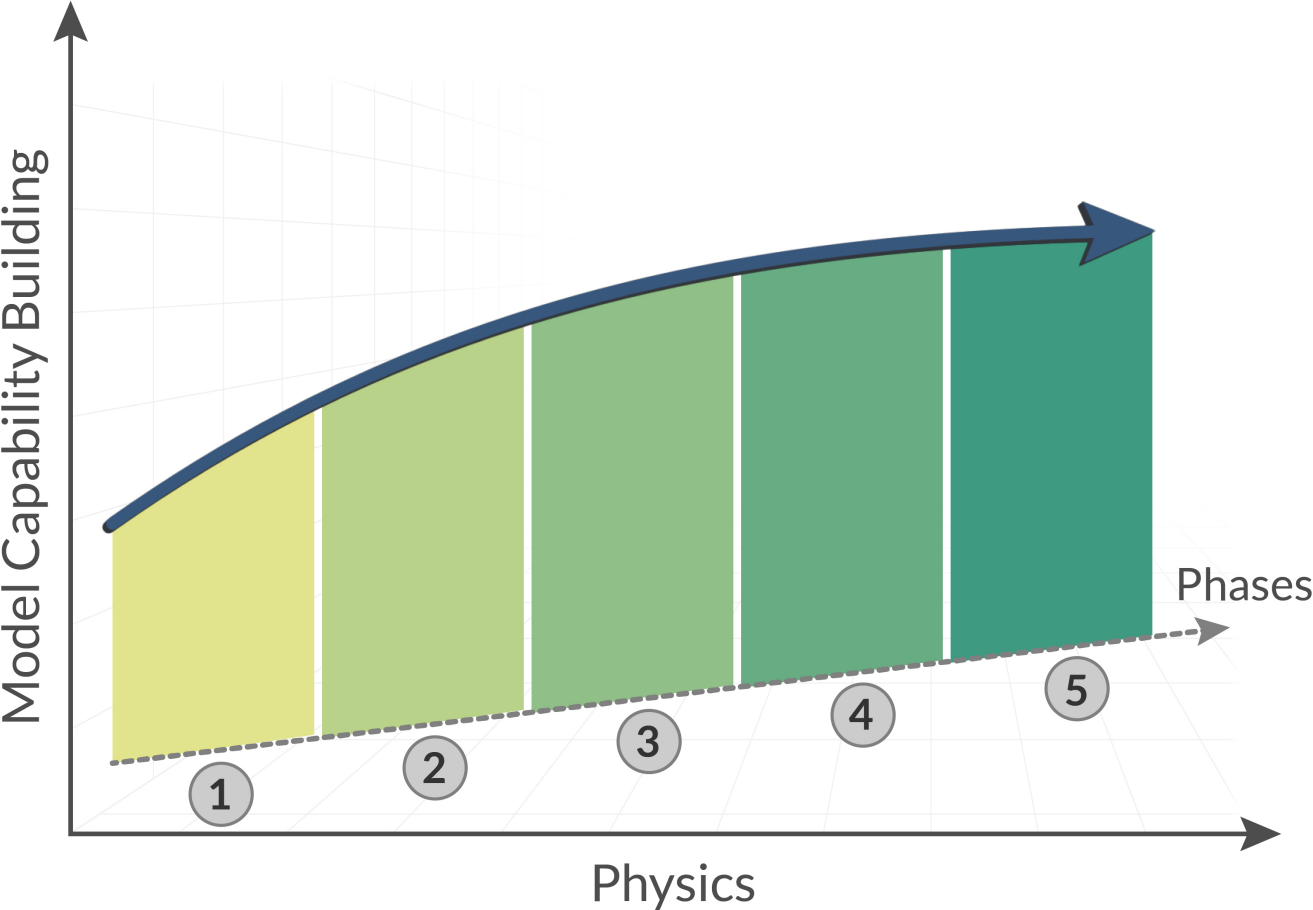
The pathway for advancing urban climate modelling capabilities and accuracy.

The pathways to advancing urban climate modelling above and below the ground involve building model capabilities to better address physical processes at the interaction zone, as well as in the atmospheric-surface layer and the subsurface layer (note that we refer to ‘layers’ to stress that only a portion of the atmosphere and subsurface are influenced by UHIs and SUHIs). While various physical processes and urban features affect urban climate at different levels, we point out five key physical processes that need to be advanced, as illustrated in figure 2, because they are largely overly simplified or even overlooked: (i) surface–subsurface heat and moisture coupling, (ii) building–vegetation resolved radiation, (iii) urban form resolved aerodynamics, (iv) ground-dependent latent heat transport to near-surface, and (v) water uptake-dependent plant transpiration.

The heat and moisture transfer in the interaction zone is arguably the most important process affecting the coupling between surface and subsurface urban heat. While in mesoscale simulations, land surface models have been progressively advanced to solve this key physical process, ground moisture representation, various types of impervious surfaces and green infrastructure are highly simplified. Efforts need to be paid to advance high-resolution representation of moisture transport, sensible heat flux on impervious surfaces and radiative fluxes due to shading for advancing modelling endeavours of surface–subsurface heat and moisture transfer couplings.

Buildings and vegetation are key elements in cities, which involve complex sensible, latent and radiative heat transfer. Their shading effect also significantly affects the incoming shortwave radiation on ground surface. To this end, modelling capability in resolving building and vegetation related radiative heat needs to be enhanced. Major buildings in cities and trees of large sizes need to be modelled with realistic geometry.

Urban aerodynamics in urban microclimate modelling have been largely resolved using Reynolds-averaged Navier–Stokes and large-eddy simulation models. The present modelling challenges lie in the representation of realistic representation of buildings, including their orientations, sizes and geometries. Buildings and streets are highly parameteried, which usually lead to unrealistic modelling result, particularly at local scale.

Latent heat transfer from ground surface to near-surface in microclimate modelling is usually overly simplified or even overlooked. Most models rely on generic soil types or empirical soil moisture datasets that are inaccurate or too coarse for heterogeneous urban soils (e.g. streets, parks, gardens). High-resolution, *in situ* measurements of ground moisture need to be carried out in cities to facilitate the calibration, validation and advancing of models.

Water uptake-dependent plant transpiration plays a critical role in urban cooling by regulating latent heat flux through vegetation. This process is strongly influenced by ground moisture availability, root depth and plant physiology. Accurately modelling transpiration requires coupling plant hydraulics with surface energy balance to capture dynamic water fluxes under varying environmental stress. Improved representation of this mechanism enhances predictions of evapotranspirative cooling, particularly under extreme heat and drought conditions in urban environments.

To enhance modelling capability over all these critical physical processes, multiscale- and multiphysics-oriented approaches are crucial. For example, while some dominant urban (micro)climate drivers, such as urban wind, usually need to be modelled at an urban morphology-resolved scale, transpiration of urban trees could be modelled as a heat sink at the tree scale. Machine learning techniques could also be used to provide emulators for parts of the physical processes that could be computationally expensive to model using computational fluid dynamics simulations.

### (c) Open research questions

Understanding and managing urban heat requires theoretical and modelling frameworks that explicitly capture the coupling between surface and subsurface processes. In addition to existing opportunities to improve the simulation of urban heat propagation above and below ground, we identify three interconnected research frontiers that remain essential for advancing characteriation, mitigation and adaptation strategies. These are: (i) refining the physical characterization of the surface–subsurface interaction zone; (ii) deepening knowledge of these couplings under current and future climates; and (iii) accelerating heat adaptation and mitigation strategies through interventions that leverage both natural and engineered solutions. For each frontier, we propose the following targeted research questions.

#### (i) Advancing characteriation and modelling of the surface–subsurface interaction zone

(a) What is the spatial extent of the interaction zone within which the heat and mass transfer processes governing the couplings between UHIs and SUHIs occur?
(b) What variables dominate the thickness of the interaction zone, the thermal response time, peak temperature variations and recovery patterns within it?
(c) How do varying tree canopy densities, root system configurations and vegetation types modify the vertical heat flux and temperature profiles at the interaction zone?
(d) How do the urban structure and morphology above and below the ground quantitatively influence the vertical heat flux and temperature profiles at the interaction zone?
(e) What modelling approaches can be adopted from the existing tools and further developed to realistically represent these coupled systems at various scales?

#### (ii) Deepening understanding of the surface–subsurface couplings in global and future climates

(a) What is the influence of different climatic zones on the surface–subsurface couplings?
(b) What is the influence of heat waves and flooding on the surface–subsurface couplings?
(c) What is the influence of polarizing urban humidity on the surface–subsurface couplings?
(d) What is the influence of rising sea levels and other hydrodynamic phenomena on the surface–subsurface couplings?
(e) What is the influence of more frequent extreme climate on the surface–subsurface couplings?

#### (iii) Underpinning the acceleration of urban heat adaptation and mitigation

(a) How can adaptation strategies be developed to address the coupled challenge of surface and subsurface overheating?
(b) What surface design interventions most effectively modulate coupled surface–subsurface heat and moisture fluxes?
(c) How can green infrastructure be optimized to manage both surface and subsurface thermal regimes?
(d) How can synergistic integration of nature-based solutions and technological interventions be designed to jointly mitigate surface and subsurface overheating?
(e) How can integrated surface–subsurface feedback inform and accelerate urban heat mitigation at scale?

Advancing the physical characteriation of the surface–subsurface interaction zone and deepening our understanding of the couplings between UHIs and SUHIs under current and future climates will require theoretical, computational and experimental efforts across relevant spatial and temporal scales. Similarly, accelerating heat adaptation and mitigation strategies through natural and engineered solutions will depend on community-informed, concrete decision-making by planners, policymakers and stakeholders, firmly grounded in a sound scientific understanding of the challenges at hand.

## 5. Closing comments and opening of the Theme Issue

This Introductory Article presents an integrated examination of the mechanisms, drivers and impacts of heat propagation in urban environments, highlighting the critical importance of understanding and addressing such phenomenon both above and below ground. Our analysis highlights that while surface UHIs have been studied for over 200 years, SUHIs have only been explored for more than 20 years at the time of writing. Importantly, studies about these phenomena have traditionally been performed in silos, focusing on either surface UHIs or SUHIs. Moreover, these studies have been customarily presented in different venues and discussed by distinct communities. As a result, the full understanding of surface UHIs and SUHIs, similar to their interactions and couplings, have remained inadequately understood, despite their marked implications for urban sustainability and resilience.

A contemporary and sound understanding of urban climates requires the consideration of the multifaceted heat and mass transfer mechanisms and phenomena operating across the interaction zone between the surface and subsurface of cities. From this perspective, integrated numerical frameworks and comprehensive experiments must bridge atmospheric and subsurface processes while maintaining feasibility. Monitoring systems must also evolve to capture the full three-dimensional nature of urban thermal anomalies. Yet, mitigation strategies must be reconceptualized to address thermal challenges holistically, considering both immediate and long-term impacts across the whole urban thermal profile. Success in these domains will require unprecedented cross-disciplinary collaboration and innovative approaches to both technical solutions and policy frameworks.

As research in this cross-disciplinary field advances, this Theme Issue gathers some of the latest scientific studies focusing on the understanding and analysis of urban heat spreading above and below ground (table 2). The studies gathered in this Theme Issue exemplify the remarkable diversity of both the fundamental and applied challenges that remain in addressing urban heat and its impacts. They also highlight the breadth of investigative methods brought to bear on these challenges, reflecting the wide spatial and temporal scales at which urban heat manifests—ranging from regions to individual households and from decades to hours or less. At the same time, the contributions point to a comparatively more advanced understanding of surface UHIs compared to their subsurface counterparts. This imbalance underscores the need for continued research across both domains, with the progress achieved about surface UHIs offering a valuable foundation for advancing knowledge of SUHIs.

**Table 2.** The selected scientific contributions to this Theme Issue.

running order	list of authors	title
1	Rotta Loria, Alessandro F.; Carmeliet, Jan; Zhao, Yongling	Urban heat above and below ground: towards improved understanding, modelling, mitigation, and adaptation (Introductory/perspective perspective)
2	Brandi, Aldo; Irani Rahaghi, Abolfazl; Zonato, Andrea; Manoli, Gabriele	Urbanization effects on lake-land circulations in complex terrain (Research article)
3	Teng, Xiaoliang; Wu, Jindong; Luo, Xiaoyu; Fan, Yifan; Ge, Jian	Similarity criteria between full scale and reduced scale models for the urban heat dome flows study under calm and stable atmospheric condition (Research article)
4	Ramamurthy, Prathap; Pena, Jean Carlos; Liu, Sarah; Gonzalez-cruz, Jorge	The interaction between urban heat island intensity and sea breeze effect (Research article)
5	Xue, Yunpeng; Zhao, Yongling; Wai, KaMing; Yuan, Chao; Carmeliet, Jan	Heat and flow dynamics in cities: an experimental comparative study across diverse urban morphologies (Research article)
6	Chu, Zhonghao; Rotta Loria, Alessandro F.	Influence of the underground urban morphology on subsurface heat islands (Research article)
7	Binder, Martin; Händel, Falk; Engelmann, Christian; Steiner, Brian; Hasler, Alma; García Gil, Alejandro; Epting, Jannis	The subsurface urban heat island of Basel-City: more than a decade of spatiotemporal high-resolution monitoring and modelling (Research article)
8	Dohmwirth, Verena; Menberg, Kathrin; Bayer, Peter; Mauder, Matthias; Blum, Philipp; Benz, Susanne	Sustainable potential of shallow geothermal heat recycling in Dresden, Germany (Research article)
9	Previati, Alberto; Gallia, Luca; Crosta, Giovanni	Impact of urbanization and climate change on underground temperatures: a modelling study in Milan (Italy) (Research article)
10	Sun, Maoran; Pan, Jiayu; Zhao, Qunshan; Bardhan, Ronita	Heat stress dichotomy: long-term adaptation and acute shock in London domestic environments (Research article)
11	Debnath, Ramit; Chandel, Taran; Han, Fengyuan; Bardhan, Ronita	Heatwave increases nighttime light intensity in hyperdense cities of the Global South: a double machine learning study (Research article)
12	Cui, Yuanfeng; Chu, Minghan; He, Zhejun; Albertson, John; Wang, Zhihua; Li, Qi	Estimating anthropogenic heat flux by assimilating meteorological observations with Kalman filter approach (Research article)
13	Houget, Tanguy; Garbero, Valeria; Piras, Marco; Dellandrea, Emmanuel; Salizzoni, Pietro	Micro-scale modelling of the urban heat island hazard during heatwaves, a case study in Turin (Research article)
14	Wang, Huanhuan; Wang, Qun; Fan, Yifan; Zhang, Yan; Wang, Xiaoxue; Li, Yuguo	Applicability of two computational fluid dynamics models and one mesoscale model for predicting urban heat island circulation (Research article)
15	Wang, Chenghao; Wang, Yihang; Wang, Zhihua; Yang, Xueli	Causal network and dynamic synchronization of urban thermal environment (Research article)
16	Lau, Kevin; Ng, Edward; Yuan, Chao	Urban heat island adaptation and mitigation in practice: lessons from policy implementation in five cities (Opinion article)

The gathered studies may be grouped into six interrelated thematic domains:

— Couplings and interactions between surface and subsurface heat islands (Article 1): Rotta Loria *et al*. [114] provide a synthesis of key drivers of UHIs and SUHIs, limitations in prevailing analysis approaches and open research questions for improving computational fidelity.
— Surface urban heat dynamics (Articles 2−5): Brandi *et al.* [115] show how urban-induced changes in heat advection can increase air temperatures over lakes or cool coastal zones, as demonstrated in the Lake Geneva region. Teng *et al*. [116] propose a new similarity criterion that links full-scale and reduced-scale heat domes over square city models, enabling more representative laboratory experiments. Ramamurthy *et al*. [117] demonstrate that in Houston, UHI effects sustain persistent hot spots even during sea breeze episodes, with secondary flows generated by sea breeze–UHI interactions. Xue *et al.* [118] investigate non-isothermal flows in varying street canyon geometries, highlighting mechanisms of heat plume generation, buoyant updraft development and canopy-layer temperature variations.
— Subsurface urban heat dynamics (Articles 6−9): Chu *et al*. [119] examine the influence of underground urban morphology on SUHIs, showing that their intensity depends strongly on underground heat source density and groundwater flow. Binder *et al*. [120] present a decade-long observational record from Basel, Switzerland, documenting groundwater temperature increases of up to 20°C due to SUHIs. Dohmwirth *et al*. [121] estimate that stored shallow geothermal heat in Dresden, Germany, could theoretically supply the city’s space heating demand for 3.5 years. Previati *et al*. [122] analye the combined impacts of urbanization and climate change on subsurface thermal regimes, underscoring the need for sustainable geothermal resource management.
— Impacts of urban heat (Articles 10−11): Sun *et al*. [123] report pronounced indoor temperature increases in London during heatwave events, pointing to elevated risks of indoor heat stress. Debnath *et al*. [124] find significant rises in nighttime light intensity during heatwaves in major Global South cities, signaling amplified nocturnal heat exposure.
— Methodological advances (Articles 12−15): Cui *et al*. [125] introduce a Kalman filter–based method for estimating anthropogenic heat fluxes, improving temporal accuracy and magnitude estimation. Houget *et al*. [126] apply multiple linear regression and convolutional neural networks to map near-surface air temperatures in Turin, Italy, achieving prediction errors of approximately 1.2 °C. Wang *et al*. [127] compare building-resolved computational fluid dynamics simulations with weather research and forecasting models, showing that the former provide greater accuracy than the latter but at higher computational cost. Wang *et al*. [128] develop a causal network analysis framework for continental-scale temperature anomalies in the United States, revealing strong spatial coherence and small-world properties, particularly at night.
— Urban policy (Article 16): Lau *et al*. [129] provide a policy-oriented perspective on embedding urban heat science into planning and regulation, emphasizing inter-agency coordination, knowledge co-production and the development of user-friendly tools.

Taken together, these studies provide a multidisciplinary foundation for enhanced understanding, analysis, mitigation and adaptation of surface (UHIs) and SUHIs. We are pleased to present this Theme Issue to the international community of researchers, practitioners, policymakers and stakeholders, with the goal of fostering multidisciplinary dialogue and accelerating progress towards resilient, climate-adaptive cities.

## Data Availability

This article has no additional data.
